# Supplementary material for: Review: Proteomic Techniques for the Development of Flood-Tolerant Soybean
Source: Int J Mol Sci. 2020 Oct 12;21(20):7497. doi: 10.3390/ijms21207497 (PMC7589014; doi:10.3390/ijms21207497)
Supplement: Supplementary file 1 [file ijms-21-07497-s001.pdf]

Review: Proteomic Techniques for the Development of Flood-Tolerant Soybean

Xin Wang and Setsuko Komatsu

Table 1. Morphological and physiological effect of flooding stress on soybean.

Traits	Growth stage/ Flooding time	Observations	Reference
plant weight	2-day-old/3, 6, 12, 24 h	Fresh weight of germinating seed increased during the first 3 h of flooding and flood slowed down soybean growth.	Yin et al., 2014a
	3-day-old/5 days	Fresh weight of plant decreased almost 50% in flooded soybean compared with untreated soybean.	Khatoon et al., 2012a
	56-day-old/14 days	Plant height was 13 to 15% shorter under flooding than control.	VanToai et al., 2001
root, hypocotyl, cotyledon, leaf	2-day-old/1, 2, 3, 4 days; 5-day flood followed by 3-day recovery	Flooding inhibited root development, caused shorter length/less pigmentation of hypocotyl, and led detachment of cotyledon.	Shi et al., 2008; Hashiguchi et al., 2009; Nanjo et al., 2014
	3-day-old/3, 6 days	Flooding reduced length of shoot/root and number of secondary roots.	Khatoon et al., 2012b
	7-day-old/1, 2, 3 days	Flooding delayed root elongation, hypocotyl elongation, and development of first leaf.	Salavati et al., 2012
	8-day-old/7 days	Dry weight of root linearly decreased with length and surface area of root under flooding.	Sakazono et al., 2014
	28-day-old/7, 14, 21 days	Flooding decreased leaf area, while enhanced development of adventitious root.	Bacanamwo and Purcell, 1999
flowering time	V3-growth-stage/21 days	Flooding did not change days to flowering, whereas it increased days to maturity.	Githiri et al., 2006
yield	V3-growth-stage/21 days	Flooding reduced number of branch/pod/seed and seed weight; however, it increased 100-seed weight.	Githiri et al., 2006
	V5-growth-stage/3, 5, 7, 9 days	Number of pod/seed decreased when flooding was prolonged and seed yield reduced with stress duration of 3 days.	Jin-Woong and Yamakawa, 2006
	R1-growth-stage/14 days	Flooding was severe on grain yield with more than 60% reduced relative to control yield.	VanToai et al., 2001
aerenchyma formation	V1-growth-stage/28 days	Secondary aerenchyma formation was morphological acclimation to flooding and it supplied oxygen to root nodules.	Shimamura et al., 2002
	V12-growth-stage/10 days	Nitrogen metabolism in nodulated-soybean root partially recovered during prolonged flooding and it was associated with aerenchyma formation.	Thomas et al., 2005
cellular processes			
cell-wall reorganization	2-day-old/1, 2, 3, 4 days	Under flooding, plasma membrane contributed to cell-wall construction, and proteins related to cell-wall lignification and synthesis were impeded.	Nanjo et al., 2013; Komatsu et al., 2009; 2010; Yin et al., 2016
calcium signaling	2-day-old/3, 6, 12, 24, 48 h	Under flooding, calcium ion was elevated in root tip/cotyledon, and calcium signaling played roles in protein folding and energy metabolism.	Komatsu et al., 2013a; Yin et al., 2014b; Wang and Komatsu, 2016; 2017

cell death	2-day-old/1, 2, 3, 4, 6 days	Flooding caused injurious effect of cell death in root.	Nanjo et al., 2013; Komatsu et al., 2013b; Oh et al., 2014; Mustafa et al., 2015; Hashimoto et al., 2020
protein metabolism	2-day-old/3, 24, 48 h	Flooding inhibited protein synthesis, glycosylation, and folding *via* ribosomes, calnexin, and PDI-like proteins; however, it activated ubiquitin-mediated proteolysis.	Yanagawa and Komatsu, 2012; Yin et al., 2016; Wang and Komatsu, 2016; Komatsu et al., 2012; Mustafa and Komatsu, 2014
RNA metabolism	2-day-old/3, 6, 24 h	Flooding suppressed mRNA processing, and RNA-regulatory related proteins of glycine-rich RNA binding protein 3 and eukaryotic aspartyl protease triggered soybean tolerance to initial stress.	Yin et al., 2016; Yin and Komatsu, 2016
energy metabolism	2-day-old/2 days	Activated fermentation *via* alcohol dehydrogenase and biotinylation-mediated energy management responded to flooding.	Komatsu et al., 2013b; Wang et al., 2016a
carbohydrate metabolism	2-day-old/3, 24 h	Flooding caused imbalance of carbohydrate metabolism, while fructose conferred soybean tolerance towards initial flooding.	Nanjo et al., 2010; Wang et al., 2017
metabolite accumulation	2-day-old/2, 4 days	Flooding accelerated accumulation of citrate, 2-oxoglutarate, aconitate, succinate, fumarate, alanine, GABA, pyruvate, NAD, and NADH; however, it decreased ATP.	Komatsu et al., 2011; Nakamura et al., 2012; Wang et al., 2018
hormone signaling	2-day-old/3, 6, 12, 24, 48, 72 h	Ethylene biosynthesis and ABA signaling mediated protein phosphorylation in initial flooding.	Yin et al., 2014a; Komatsu et al., 2013c; Yin and Komatsu, 2015; Wang et al., 2016b

ABA, abscisic acid; GABA, gamma-aminobutyric acid; PDI, protein disulfide isomerase.

References in Table S1

Bacanamwo, M.; Purcell, L.C. Soybean root morphological and anatomical traits associated with acclimation to flooding. *Crop Sci.* **1999**, *39*, 143–149.

Githiri, S.M.; Watanabe, S.; Harada, K.; Takahashi, R. QTL analysis of flooding tolerance in soybean at an early vegetative growth stage. *Plant Breed.* **2006**, *125*, 613–618.

Hashiguchi, A.; Sakata, K.; Komatsu, S. Proteome analysis of early-stage soybean seedlings under flooding stress. *J. Proteome Res.* **2009**, *8*, 2058–2069.

Hashimoto, T.; Mustafa, G.; Nishiuchi, T.; Komatsu, S. Comparative analysis of the effect of inorganic and organic chemicals with silver nanoparticles on soybean under flooding stress. *Int. J. Mol. Sci.* **2020**, *21*, 1300.

Jin-Woong, C.H.O.; Yamakawa, T. Effects on growth and seed yield of small seed soybean cultivars of flooding conditions in paddy field. *J. Fac. Agr. Kyushu Univ*, **2006**, *51*, 189–193.

Khatoon, A.; Rehman, S.; Hiraga, S.; Makino, T.; Komatsu, S. Organ-specific proteomics analysis for identification of response mechanism in soybean seedlings under flooding stress. *J. Proteomics* **2012a**, *75*, 5706–5723.

Khatoon, A.; Rehman, S.; Oh, M.W.; Woo, S.H.; Komatsu, S. Analysis of response mechanism in soybean under low oxygen and flooding stresses using gel-base proteomics technique. *Mol. Biol. Rep.* **2012b**, *39*, 10581–10594.

Komatsu, S.; Wada, T.; Abaléa, Y.; Nouri, M.Z.; Nanjo, Y.; Nakayama, N.; Shimamura, S.; Yamamoto, R.; Nakamura, T.; Furukawa, K. Analysis of plasma membrane proteome in soybean and application to flooding stress response. *J. Proteome Res.* **2009**, *8*, 4487–4499.

Komatsu, S.; Kobayashi, Y.; Nishizawa, K.; Nanjo, Y.; Furukawa, K. Comparative proteomics analysis of differentially expressed proteins in soybean cell wall during flooding stress. *Amino Acids* **2010**, *39*, 1435–1449.

Komatsu, S.; Yamamoto, A.; Nakamura, T.; Nouri, M.Z.; Nanjo, Y.; Nishizawa, K.; Furukawa, K. Comprehensive analysis of mitochondria in roots and hypocotyls of soybean under flooding stress using proteomics and metabolomics techniques. *J. Proteome Res.* **2011**, *10*, 3993–4004.

Komatsu, S.; Kuji, R.; Nanjo, Y.; Hiraga, S.; Furukawa, K. Comprehensive analysis of endoplasmic reticulum-enriched fraction in root tips of soybean under flooding stress using proteomics techniques. *J. Proteomics* **2012**, *77*, 531–560.

Komatsu, S.; Makino, T.; Yasue, H. Proteomic and biochemical analyses of the cotyledon and root of flooding-stressed soybean plants. *PLoS One* **2013a**, *8*, e65301.

Komatsu, S.; Nanjo, Y.; Nishimura, M. Proteomic analysis of the flooding tolerance mechanism in mutant soybean. *J. Proteomics* **2013b**, *79*, 231–250.

Komatsu, S.; Han, C.; Nanjo, Y.; Altaf-Un-Nahar, M.; Wang, K.; He, D.; Yang P. Label-free quantitative proteomic analysis of abscisic acid effect in early-stage soybean under flooding. *J. Proteome Res.* **2013c**, *12*, 4769–4784.

Mustafa, G.; Komatsu, S. Quantitative proteomics reveals the effect of protein glycosylation in soybean root under flooding stress. *Front. Plant Sci.* **2014**, *5*, 627.

Mustafa, G.; Sakata, K.; Komatsu S. Proteomic analysis of flooded soybean root exposed to aluminum oxide nanoparticles. *J. Proteomics* **2015**, *128*, 280–297.

Nakamura, T.; Yamamoto, R.; Hiraga, S.; Nakayama, N.; Okazaki, K.; Takahashi, H.; Uchimiya, H.; Komatsu, S. Evaluation of metabolite alteration under flooding stress in soybeans. *JARQ-Jpn. Agric. Res. Q.* **2012**, *46*, 237–248.

Nanjo, Y.; Jang, H.Y.; Kim, H.S.; Hiraga, S.; Woo, S.H.; Komatsu, S. Analyses of flooding tolerance of soybean varieties at emergence and varietal differences in their proteomes. *Phytochemistry* **2014**, *106*, 25–36.

Nanjo, Y.; Nakamura, T.; Komatsu, S. Identification of indicator proteins associated with flooding injury in soybean seedlings using label-free quantitative proteomics. *J. Proteome Res.* **2013**, *12*, 4785–4798.

Nanjo, Y.; Skultety, L.; Ashraf, Y.; Komatsu, S. Comparative proteomic analysis of early-stage soybean seedlings responses to flooding by using gel and gel-free techniques. *J. Proteome Res.* **2010**, *9*, 3989–4002.

Oh, M.; Nanjo, Y.; Komatsu, S. Gel-free proteomic analysis of soybean root proteins affected by calcium under flooding stress. *Front. Plant Sci.* **2014**, *5*, 559.

Sakazono, S.; Nagata, T.; Matsuo, R.; Kajihara, S.; Watanabe, M.; Ishimoto, M.; Shimamura, S.; Harada, K.; Takahashi, R.; Mochizuki, T. Variation in root development response to flooding among 92 soybean lines during early growth stages. *Plant Prod. Sci.* **2014**, *17*, 228–236.

Salavati, A.; Khatoon, A.; Nanjo, Y.; Komatsu, S. Analysis of proteomic changes in roots of soybean seedlings during recovery after flooding. *J. Proteomics* **2012**, *75*, 878–893.

Shi, F.; Yamamoto, R.; Shimamura, S.; Hiraga, S.; Nakayama, N.; Nakamura, T.; Yukawa, K.; Hachinohe, M.; Matsumoto, H.; Komatsu, S. Cytosolic ascorbate peroxidase 2 (cAPX 2) is involved in the soybean response to flooding. *Phytochemistry* **2008**, *69*, 1295–1303.

Shimamura, S.; Mochizuki, T.; Nada, Y.; Fukuyama, M. Secondary aerenchyma formation and its relation to nitrogen fixation in root nodules of soybean plants (*Glycine max*) grown under flooded conditions. *Plant Prod. Sci.* **2002**, *5*, 294–300.

Thomas, A.L.; Guerreiro, S.M.; Sodek, L. Aerenchyma formation and recovery from hypoxia of the flooded root system of nodulated soybean. *Ann. Bot.* **2005**, *96*, 1191–1198.

VanToai, T.T.; St. Martin, S.K.; Chase, K.; Boru, G.; Schnipke, V.; Schmitthenner, A. F.; Lark, K.G. Identification of a QTL associated with tolerance of soybean to soil waterlogging. *Crop Sci.* **2001**, *41*, 1247–1252.

Wang, X.; Komatsu, S. Gel-free/label-free proteomic analysis of endoplasmic reticulum proteins in soybean root tips under flooding and drought stresses. *J. Proteome Res.* **2016**, *15*, 2211–2227.

Wang, X.; Komatsu, S. Proteomic analysis of calcium effects on soybean root tip under flooding and drought stresses. *Plant Cell Physiol.* **2017**, *58*, 1405–1420.

Wang, X.; Oh, M.; Sakata, K.; Komatsu, S. Gel-free/label-free proteomic analysis of root tip of soybean over time under flooding and drought stresses. *J. Proteomics* **2016a**, *130*, 42–55.

Wang, X.; Oh, M.; Komatsu, S. Characterization of *S*-adenosylmethionine synthetases in soybean under flooding and drought stresses. *Biol. Plantarum* **2016b**, *60*, 269–278.

Wang, X.; Sakata, K.; Komatsu, S. An integrated approach of proteomics and computational genetic modification effectiveness analysis to uncover the mechanisms of flood tolerance in soybeans. *Int. J. Mol. Sci.* **2018**, *19*, 1301.

Wang, X.; Zhu, W.; Hashiguchi, A.; Nishimura, M.; Tian, J.; Komatsu, S. Metabolic profiles of flooding-tolerant mechanism in early-stage soybean responding to initial stress. *Plant Mol. Biol.* **2017**, *94*, 669–685.

Yanagawa, Y.; Komatsu, S. Ubiquitin/proteasome-mediated proteolysis is involved in the response to flooding stress in soybean roots, independent of oxygen limitation. *Plant Sci.* **2012**, *185*, 250–258.

Yin, X.; Komatsu, S. Nuclear proteomics reveals the role of protein synthesis and chromatin structure in root tip of soybean during the initial stage of flooding stress. *J. Proteome Res.* **2016**, *15*, 2283–2298.

Yin, X.; Komatsu, S. Quantitative proteomics of nuclear phosphoproteins in the root tip of soybean during the initial stages of flooding stress. *J. Proteomics* **2015**, *119*, 183–195.

Yin, X.; Nishimura, M.; Hajika, M.; Komatsu, S. Quantitative proteomics reveals the flooding-tolerance mechanism in mutant and abscisic acid-treated soybean. *J. Proteome Res.* **2016**, *15*, 2008–2025.

Yin, X.; Sakata, K.; Komatsu, S. Phosphoproteomics reveals the effect of ethylene in soybean root under flooding stress. *J. Proteome Res.* **2014a**, *13*, 5618−5634.
Yin, X.; Sakata, K.; Nanjo, Y.; Komatsu, S. Analysis of initial changes in the proteins of soybean root tip under flooding stress using gel-free and gel-based proteomic techniques. *J. Proteomics* **2014b**, *106*, 1−16.

Table 2. Plant-omic analysis to understand flood-response mechanisms on soybean.

Experimental materials	Growth stage/ Flooding time	Findings	Reference
Proteomics			
total cellular			
root tip, root, hypocotyl	2-day-old/3, 6, 12, 24 h	Initial flooding caused imbalanced accumulation of proteins involved in carbohydrate metabolism, while calcium-signal transduction protected soybean from stress.	Yin et al., 2014a; Nanjo et al., 2010
	2-day-old/2, 5 days	Under flooding, phosphatase 2A subunit-like proteins played roles on cell death in root tip, while isoflavone reductase and disease-related proteins were responsible for suppressed growth of soybean seedling.	Nanjo et al., 2013; Khatoon et al., 2012a
	14-day-old/3, 7 days	Soybean coped with waterlogging *via* regulation of carbohydrate consumption and programmed cell death.	Alam et al., 2010
leaf	6-day-old/2, 4, 6, 8 days	Waterlogging impaired metabolisms of photosynthesis/photorespiration and hydroxypyruvate reductase limited rate of photorespiration in soybean leaf.	Oskuei et al., 2017
root	2-day-old/3-day flood followed by 7-day recovery; 2-, 4-day flood followed by 4-, 6-, 8-day	Peroxidases and proteins related to lignification/cytoskeletal organization played roles in post-flooding recovery.	Salavati et al., 2012; Khan et al., 2014
	3-day-old/3, 6 days	Decreased proteins involved in destination and defense were associated with suppression of soybean growth under flooding.	Khatoon et al., 2012b
hypocotyl	2-day-old/2-day-flood followed by 2- and 4-day recovery	Activated glycosylation and secondary metabolism enabled soybean recovery from flooding-induced damage.	Khan et al., 2015
subcellular proteins			
cell wall/root, hypocotyl	2-day-old/2 days	Decreased lipoxygenases, germin-like protein precursors, glycoprotein precursors, and super oxide dismutase, leading to suppressed lignification *via* inhibited biosynthesis of ROS and JA under flooding.	Komatsu et al., 2010
nuclei/root tip, root	2-day-old/3 h	Initial flooding suppressed protein translation, and decreased zinc finger, glycine-rich protein, and rRNA-processing protein were affected by ABA-mediated phosphorylation.	Yin and Komatsu, 2015; 2016
	2-day-old/2 days	Under flooding, increased RACK1 and poly-ADP-ribosylation responded to stress; however, decreased zinc finger proteins, cell division cycle 5, and transduction played roles in tolerance.	Komatsu et al.,2013;2014; Oh et al., 2014
mitochondria/root, hypocotyl	2-day-old/2 days	Flooding impaired electron-transport chains, and proteins involved in TCA cycle, glutathione, and aldehyde metabolism played roles in oxidation and peroxide scavenging.	Komatsu et al., 2011; Kamal and Komatsu, 2015

ER/root tip	2-day-old/2 days	Flooding suppressed protein synthesis in the ER and caused dysfunction of protein folding, leading to reduction of glycoproteins.	Wang and Komatsu, 2016; Komatsu et al., 2012
post-translational modifications			
phosphorylation/root tip, root, hypocotyl	2-day-old/3, 12, 24 h	Flooding altered phosphorylation status of proteins related to ethylene signaling, ABA response, protein folding, cell construction, and ATP generation.	Yin et al., 2014b; Yin and Komatsu, 2015; Nanjo et al., 2010; 2012
glycosylation/root tip, root	2-day-old/2 days	Flooding reduced glycoproteins, and glycosylation status of proteins related to stress, glycolysis, and protein degradation were predominantly affected.	Wang and Komatsu, 2016; Mustafa and Komatsu, 2014
nitrosylation/root	2-day-old/3, 9, 24 h	*S*-nitrosylation status of proteins involved in sugar-degrading contributed to anaerobic shift under flooding.	Hashiguchi and Komatsu, 2018
ubiquitination/root	2-day-old/2 days	Accumulation of CSN proteins enhanced degradation of ubiquitinated proteins under flooding.	Yanagawa and Komatsu, 2012
transcriptomics/root, hypocotyl, cotyledon, leaf	2-day-old/6, 12 h/root including hypocotyl	Functional disorder of acclimative response to flooding *via* transcriptional and posttranscriptional regulations was involved in flooding injury to soybean.	Nanjo et al., 2011
	4-day-old/10 days	Constitutive basal levels of transcripts related to sugar metabolism/fermentation were sufficient to breakdown carbohydrate reserves in hypocotyl and cotyledon of soybean under submergence.	Tamang et al., 2014
	V4-growth-stage/7 days	Alterations of genes involved in cell-wall precursor and starch/sugar metabolism served as adaptive response for soybean survival from flooding.	Chen et al., 2016
metabolomics/root, hypocotyl	2-day-old/2, 4 days	Flooding accelerated accumulation of citrate, succinate, aconitate, GABA, pyruvate, NAD, NADH, and alanine.	Komatsu et al., 2011; Nakamura et al., 2012
bioinformatics/seedling	2-day-old/2-7 days; flood for 2 or 4 days followed by 6-day recovery	Soybean Proteome Database was originally constructed based on gel-based proteomics, and it updated with integrated gel-free proteomics, genomics, transcriptomics as well as metabolomics.	Sakata et al., 2009; Ohyanagi et al., 2012; Komatsu et al., 2017

ABA, abscisic acid; CSN, COP9 signalosome; ER, endoplasmic reticulum; GABA, gamma-aminobutyric acid; JA, jasmonic acid; PTM, post translational modification; RACK1, receptor for activated protein kinase C1; ROS, reactive oxygen species; TCA, tricarboxylic acid. Data have been collected from 2010 to 2020.

References in Table S2

Alam, I.; Lee, D.G.; Kim, K.H.; Park, C.H.; Sharmin, S.A.; Lee, H.; Oh, K.W.; Yun, B.W.; Lee, B.H. Proteome analysis of soybean roots under waterlogging stress at an early vegetative stage. *J. Biosci.* **2010**, *35*, 49–62.

Chen, W.; Yao, Q.; Patil, G.B.; Agarwal, G.; Deshmukh, R.K.; Lin, L.; Wang, B.; Wang, Y.; Prince, S.J.; Song, L.; Xu, D.; An, Y.C.; Valliyodan, B.; Varshney, R.K.; Nguyen, H.T. Identification and comparative analysis of

differential gene expression in soybean leaf tissue under drought and flooding stress revealed by RNA-Seq. *Front. Plant. Sci.* **2016**, *7*, 1044.

Hashiguchi, A.; Komatsu, S. Early changes in *S*-nitrosoproteome in soybean seedling under flooding stress. *Plant Mol. Biol. Rep.* **2018,** *36*, 822-831.

Kamal, A.H.; Komatsu, S. Involvement of reactive oxygen species and mitochondrial proteins in biophoton emission in roots of soybean plants under flooding stress. *J. Proteome Res.* **2015**, *14*, 2219–2236.

Khan, M.N.; Sakata, K.; Hiraga, S.; Komatsu, S. Quantitative proteomics reveals that peroxidases play key roles in post-flooding recovery in soybean roots. *J. Proteome Res.* **2014**, *13*, 5812–5828.

Khan, M.N.; Sakata, K.; Komatsu, S. Proteomic analysis of soybean hypocotyl during recovery after flooding stress. *J. Proteomics* **2015**, *121*, 15–27.

Khatoon, A.; Rehman, S.; Hiraga, S.; Makino, T.; Komatsu, S. Organ-specific proteomics analysis for identification of response mechanism in soybean seedlings under flooding stress. *J. Proteomics* **2012a**, *75*, 5706–5723.

Khatoon, A.; Rehman, S.; Oh, M.W.; Woo, S.H.; Komatsu, S. Analysis of response mechanism in soybean under low oxygen and flooding stresses using gel-base proteomics technique. *Mol. Biol. Rep.* **2012b**, *39*, 10581–10594.

Komatsu, S.; Han, C.; Nanjo, Y.; Altaf-Un-Nahar, M.; Wang, K.; He, D.; Yang P. Label-free quantitative proteomic analysis of abscisic acid effect in early-stage soybean under flooding. *J. Proteome Res.* **2013**, *12*, 4769–4784.

Komatsu, S.; Hiraga, S.; Nouri, M.Z. Analysis of flooding-responsive proteins localized in the nucleus of soybean root tips. *Mol. Biol. Rep.* **2014**, *41*, 1127–1139.

Komatsu, S.; Kobayashi, Y.; Nishizawa, K.; Nanjo, Y.; Furukawa, K. Comparative proteomics analysis of differentially expressed proteins in soybean cell wall during flooding stress. *Amino Acids* **2010**, *39*, 1435–1449.

Komatsu, S.; Kuji, R.; Nanjo, Y.; Hiraga, S.; Furukawa, K. Comprehensive analysis of endoplasmic reticulum-enriched fraction in root tips of soybean under flooding stress using proteomics techniques. *J. Proteomics* **2012**, *77*, 531–560.

Komatsu, S.; Wang, X.; Yin, X.; Nanjo, Y.; Ohyanagi, H.; Sakata, K. Integration of gel-based and gel-free proteomic data for functional analysis of proteins through Soybean Proteome Database. *J. Proteomics* **2017**, *163*, 52–66.

Komatsu, S.; Yamamoto, A.; Nakamura, T.; Nouri, M.Z.; Nanjo, Y.; Nishizawa, K.; Furukawa, K. Comprehensive analysis of mitochondria in roots and hypocotyls of soybean under flooding stress using proteomics and metabolomics techniques. *J. Proteome Res.* **2011**, *10*, 3993–4004.

Mustafa, G.; Komatsu, S. Quantitative proteomics reveals the effect of protein glycosylation in soybean root under flooding stress. *Front. Plant Sci.* **2014**, *5*, 627.

Nakamura, T.; Yamamoto, R.; Hiraga, S.; Nakayama, N.; Okazaki, K.; Takahashi, H.; Uchimiya, H.; Komatsu, S. Evaluation of metabolite alteration under flooding stress in soybeans. *JARQ-Jpn. Agric. Res. Q.* **2012**, *46*, 237–248.

Nanjo, Y.; Maruyama, K.; Yasue, H.; Yamaguchi-Shinozaki, K.; Shinozaki, K.; Komatsu, S. Transcriptional responses to flooding stress in roots including hypocotyl of soybean seedlings. *Plant Mol. Biol.* **2011**, *77*, 129–144.

Nanjo, Y.; Nakamura, T.; Komatsu, S. Identification of indicator proteins associated with flooding injury in soybean seedlings using label-free quantitative proteomics. *J. Proteome Res.* **2013**, *12*, 4785–4798.

Nanjo, Y.; Skultety, L.; Ashraf, Y.; Komatsu, S. Comparative proteomic analysis of early-stage soybean seedlings responses to flooding by using gel and gel-free techniques. *J. Proteome Res.* **2010**, *9*, 3989–4002.

Nanjo, Y.; Skultety, L.; Uváčková, L.; Klubicová, K.; Hajduch, M.; Komatsu, S. Mass spectrometry-based analysis of proteomic changes in the root tips of flooded soybean seedlings. *J. Proteome Res.* **2012**, *11*, 372–385.

Oh, M.; Nanjo, Y.; Komatsu, S. Identification of nuclear proteins in soybean under flooding stress using proteomic technique. *Protein Pept. Lett.* **2014**, *21*, 458–467.

Ohyanagi, H.; Sakata, K.; Komatsu, S. Soybean Proteome Database 2012: update on the comprehensive data repository for soybean proteomics. *Front. Plant. Sci.* **2012**, *3*, 110.

Oskuei, B.K.; Yin, X.; Hashiguchi, A.; Bandehagh, A.; Komatsu, S. Proteomic analysis of soybean seedling leaf under waterlogging stress in a time-dependent manner. *Biochim. Biophys. Acta. Proteins Proteom.* **2017**, *1865*, 1167–1177.

Sakata, K.; Ohyanagi, H.; Nobori, H.; Nakamura, T.; Hashiguchi, A.; Nanjo, Y.; Mikami, Y.; Yunokawa, H.; Komatsu, S. Soybean proteome database: a data resource for plant differential omics. *J. Proteome Res.* **2009**, *8*, 3539-48.

Salavati, A.; Khatoon, A.; Nanjo, Y.; Komatsu, S. Analysis of proteomic changes in roots of soybean seedlings during recovery after flooding. *J. Proteomics* **2012**, *75*, 878–893.

Tamang, B.G.; Magliozzi, J.O.; Maroof, M.A.; Fukao, T. Physiological and transcriptomic characterization of submergence and reoxygenation responses in soybean seedlings. *Plant Cell Environ.* **2014**, *37*, 2350–2365.

Wang, X.; Komatsu, S. Gel-free/label-free proteomic analysis of endoplasmic reticulum proteins in soybean root tips under flooding and drought stresses. *J. Proteome Res.* **2016**, *15*, 2211–2227.

Yanagawa, Y.; Komatsu, S. Ubiquitin/proteasome-mediated proteolysis is involved in the response to flooding stress in soybean roots, independent of oxygen limitation. *Plant Sci.* **2012**, *185*, 250–258.

Yin, X.; Komatsu, S. Nuclear proteomics reveals the role of protein synthesis and chromatin structure in root tip of soybean during the initial stage of flooding stress. *J. Proteome Res.* **2016**, *15*, 2283–2298.
Yin, X.; Komatsu, S. Quantitative proteomics of nuclear phosphoproteins in the root tip of soybean during the initial stages of flooding stress. *J. Proteomics* **2015**, *119*, 183–195.
Yin, X.; Sakata, K.; Nanjo, Y.; Komatsu, S. Analysis of initial changes in the proteins of soybean root tip under flooding stress using gel-free and gel-based proteomic techniques. *J. Proteomics* **2014a**, *106*, 1–16.
Yin, X.; Sakata, K.; Komatsu, S. Phosphoproteomics reveals the effect of ethylene in soybean root under flooding stress. *J. Proteome Res.* **2014b**, *13*, 5618–5634.
